# Ankle Kinematics and Temporal Gait Characteristics over the Duration of a 6-Minute Walk Test in People with Multiple Sclerosis Who Experience Foot Drop

**DOI:** 10.1155/2018/1260852

**Published:** 2018-07-02

**Authors:** Marietta L. van der Linden, Georgia Andreopoulou, Judy Scopes, Julie E. Hooper, Thomas H. Mercer

**Affiliations:** ^1^Centre for Health, Activity and Rehabilitation Research, Queen Margaret University, Edinburgh EH21 6UU, UK; ^2^NHS Lothian, Edinburgh, UK

## Abstract

Foot drop is a common gait impairment in people with MS (pwMS) but in some foot drop may only occur after a period of prolonged walking and may be a sign of motor fatigability. The purpose of the study was to explore whether, for pwMS, an adapted six-minute walk test (6minWT) would result in an increase in foot drop as measured using electrogoniometry. Sagittal ankle kinematics were recorded for fifteen participants (10 females and 5 males, aged 37-64) with MS (EDSS 4-6) throughout the 6minWT. Ankle kinematics and temporal stride parameters were compared between the first and last 10 gait cycles of the 6minWT. Peak dorsiflexion in swing was significantly reduced at the end of the 6minWT compared to the start, with six of the fifteen participants having a decrease of two degrees or more. Statistically significant changes in temporal stride parameters suggested a decrease in walking speed. Our results suggest that with the protocol used in this study it is feasible to identify patients who experience increased foot drop as a result of a prolonged exercise task.

## 1. Introduction

Multiple sclerosis (MS) is an autoimmune disease characterized by inflammation and progressive destruction of the central nervous system (CNS). The disease usually presents between the ages of 20-40 and affects around 2.3 million people worldwide [1]. Although symptom manifestation can vary considerably among individuals, the cluster of symptoms comprising fatigue, weakness, posture, and movement disturbances is common [2]. Heesen et al. [3] reported that gait function was most frequently rated as the most important domain by people with MS (pwMS). Both the prevalence and severity of gait impairments have been found to correlate with the duration of the disease, although some studies have identified walking characteristics that are affected in people with mild MS [4, 5]. A common gait symptom is foot drop, which is a decrease in ankle dorsiflexion during the swing phase of gait, resulting in an increased likelihood for tripping and falling. Foot drop can be caused by peripheral muscle weakness of the dorsiflexor muscles, increased tone of the plantar flexor muscles, or impaired neural control causing cocontraction of agonist and antagonist muscles [6]. Particularly in pwMS foot drop may also be a sign of increased motor fatigability [7]. In this case, foot drop may not manifest itself when the patient is performing a short walk test (e.g., 10 meters) in the clinic but will only occur after a certain period of prolonged walking the duration of which varies among individuals. In a qualitative study on the experiences of assistive technology (Functional Electrical Stimulation and Ankle Foot Orthosis) to treat foot drop, some participants commented that they only used these when going out for longer walks [8].

As a consequence, this type of foot drop which only occurs occasionally or worsens as a result of prolonged exercise and is likely to be a sign of motor fatigability may be difficult to diagnose in short duration walking tests. Similarly, for some patients the benefits of therapy or assistive technology to treat foot drop may only be evident over longer duration walking tests.

Kluger et al. [9] described fatigability as an exercise-induced reduction in the ability of muscles to produce force or power, regardless of whether a task can be sustained. Several authors have suggested that this reduced performance over time is partly of central origin [10–13] and could be a compensatory mechanism for the effects of demyelination on conduction in motor pathways in people with MS.

Severijns et al. [14] reviewed the protocols and outcome measures used to study or detect motor fatigability in pwMS. They concluded that motor fatigability, assessed either at International Classification of Functioning, Disability and Health (ICF) body function level (e.g., strength) or at the ICF activity level (e.g., walking performance), is increased in most pwMS.

Previous studies evaluating motor fatigability on ICF activity level have done so by measuring the decrease in walking speed over the duration of a set walking test ranging from 6 to 12 minutes or a specified distance, e.g., [15–18]. However, only a few research groups have investigated fatigability on the ICF activity level by quantifying the deterioration in gait kinematics [19–22]. Computerised 3D motion analysis is able to detect even small changes in gait kinematics that cannot be observed visually. Unfortunately however, this technique can be impractical for routine clinical practice due to issues associated with equipment cost and need for specialist staff to collect and interpret 3D data. Flexible electrogoniometry (EGM) has been proposed as a valid alternative method by which to record gait kinematics [23, 24] in a clinical environment.

The primary aim of this pilot study was therefore to determine whether an adapted 6-minute walking test (6minWT) protocol combined with electrogoniometry could be used to detect a decrease in ankle dorsiflexion during swing suggesting motor fatigability.

As such, this study will indicate whether the protocol used in this study may be used to detect foot drop due to motor fatigability in a clinical environment. The secondary aim of this study was to assess whether temporal stride parameters (cadence and stance phase duration) and the rate of perceived exertion (RPE) change over the duration of 6 minutes of walking.

## 2. Materials and Methods

The experiment took place in a single visit to either the outpatient physiotherapy clinic or the university sports hall. The study was approved by the research ethics committees of the United Kingdom National Health Service (NHS) and Queen Margaret University. Participants provided informed consent prior to taking part in the study in accordance with the declaration of Helsinki.

### 2.1. Participants

Participants with a clinical diagnosis of definite MS according to the McDonald criteria [25] were included in the study if they presented with unilateral foot drop due to MS as diagnosed by an experienced specialist physiotherapist. The exclusion criteria were fixed ankle deformities, allergy to tape, and being unable to walk for at least two minutes without stopping (with or without walking aids). Patients with other conditions affecting their walking ability such as cardiopulmonary disease, severe visual loss and arthritis, and particularly conditions which possibly could result into foot drop such as peripheral neuropathies and disorders of the spine were also excluded.

Participants were recruited from an outpatient physiotherapy clinic.

### 2.2. Procedure/Outcome Measures

Prior to their walking assessment, all participants completed the MS walking scale (MSWS-12) which is a patient-reported outcome measure of perceived walking ability. It contains 12 questions/items with the original scoring providing 1-5 points for each item, with ‘1' meaning ‘no limitation' and ‘5' meaning ‘extreme limitation'. A total score is generated and reported on a 0 to 100 scale [26] with a higher score indicating a higher reported limitation of walking ability.

**Figure 1:**
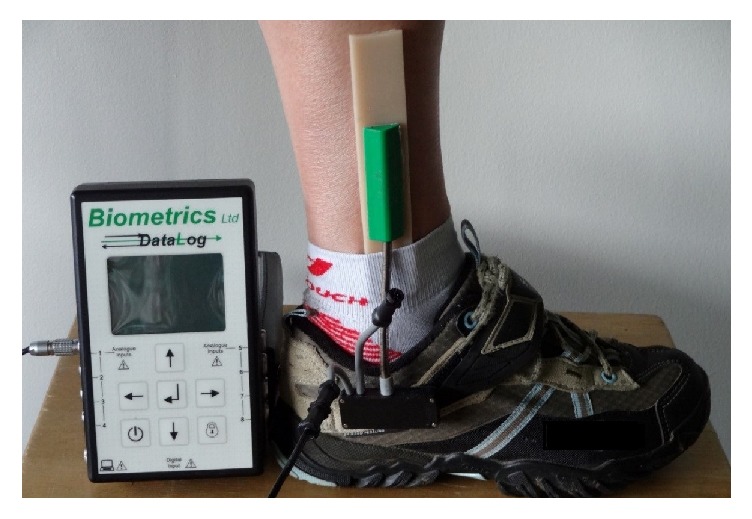
Data logger and position of the electrogoniometer end blocks on the lateral surface of the lower leg and shoe.

The two end blocks of the ankle electrogoniometer (SG110A, Biometrics Ltd., Newport, UK) were attached to the lower leg and shoe using skin-friendly double sided adhesive tape according to the manufacturer's instructions (see Figure 1). The electrogoniometer was attached to the leg for which the participant received treatment for foot drop. A pressure sensitive sensor (‘foot switch') was attached to the sole of the participant's heel to record the instant of heel contact, which allows the identification of the gait cycle (heel contact to ipsilateral heel contact).

Both the electrogoniometer and the foot switch were connected to a data logger which recorded the ankle kinematics and foot switch data synchronously at 100 Hz. During the assessment the participant carried the data logger in a waist belt.

In an adapted 6-minute walking test protocol, participants were instructed to walk around a rectangular course of 32 metres in length (10m long and 6m wide) and cover as much distance as possible over a period of 6 minutes. The number of laps were counted and the position of the participant at the end of the 6minWT was marked on the floor with tape, allowing the calculation of the total distance walked. Participants were informed that they could stop and rest if they felt they needed to. Participants were asked to wear their usual flat-soled walking shoes or trainers and removed their AFO or switched off their FES device for the duration of the test.

At every minute after the start of the 6minWT, the participant was asked to rate his or her perceived exertion using the Borg Rate of Perceived Exertion (Borg RPE) scale which is a 15-point rating scale ranging from 6 to 20, with 6 ‘no exertion' to 20 ‘maximal exertion' [27].

### 2.3. Data Analysis

**Figure 2:**
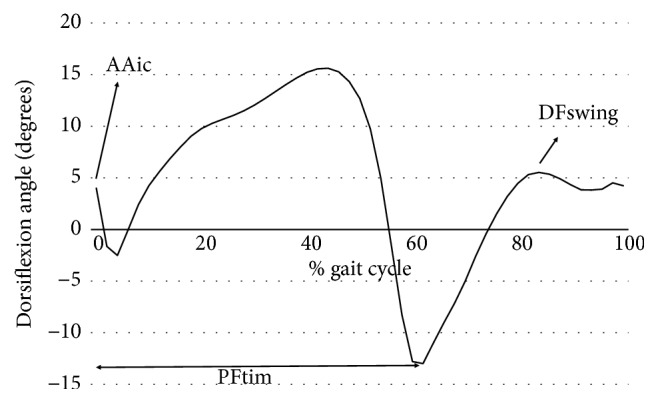
Typical sagittal ankle joint angle over one complete gait cycle showing the outcome measures AAIC (ankle angle at initial contact), DFswing (peak dorsiflexion in swing), and the timing of peak plantar flexion (PFtim), reported as % of gait cycle.

Data analysis was performed using custom-written scripts in Matlab v7.6.0 (Mathworks, Natick MA, USA). The following gait parameters were derived from the electrogoniometry and foot switch data: peak dorsiflexion during swing (DFswing), ankle angle at initial contact (AAic), cadence (steps/min), and the instant of the peak plantar flexion just before toe-off (PFtim) calculated as the percentage of the duration of the gait cycle (Figure 2). PFtim was used as an approximation of the duration of the stance phase as the instant of toe-off occurs just after PFtim [28]. All gait parameters were determined for each of the first and last 10 gait cycles of the 6minWT for each participant.

### 2.4. Statistical Analysis

Normality of the gait data was confirmed by visual inspection of the q-q plots and box plots and the Shapiro-Wilk test. Differences between gait characteristics in the first and last 10 gait cycles of the 6minWT were analysed using paired t-tests. For the RPE the values reported in the 1^st^ minute of the 6minWT were compared to those in the last minute of the 6minWT. A p-value of less than 0.05 was regarded as statistically significant. Statistical analysis was performed using SPSS v21 (IBM Software, Armonk NY, USA).

## 3. Results

Fifteen participants were recruited and attended the QMU sports hall or outpatient physiotherapy clinic for one single assessment. Participants' descriptive characteristics are presented in Table 1. Three of the fifteen participants paused for a maximum of 5 minutes because mobility issues such as lower limb weakness during the 6minWT but all completed the test.

The results for the gait characteristics and RPE are shown in Table 2. Compared to the start of the 6minWT, there was an average decrease of 1.4 and 1.6 degrees at the end of 6minWT for the AAic and DFswing, respectively, but this was only statistically significant for DFswing (p=0.03).

If analysing the results of only those who did not stop during the 6minWT (n=12), the average decrease over the duration of the 6minWT for the AAic and DFswing was less than for the whole sample; 1 and 1.4 degrees, respectively, and neither decrease was statistically significant.

Cadence was significantly reduced at the end of the 6minWT compared to the start (p<0.001). The timing of the peak plantar flexion as a percentage of the duration of the gait cycle used as a proxy for stance phase duration was also significantly later (p=0.02) at the end of the 6minWT. Both a reduced cadence and a longer stance phase duration are associated with a decrease in walking speed.

Finally, the RPE in the sixth minute of the 6minWT was significantly higher than the RPE after one minute (12.1 versus 7.9, p<0.001)

The results at an individual level (averaged over 10 gait cycles) can be seen in Table 3. Six of the 15 participants had a decrease in dorsiflexion in swing of two degrees or more [29] (average 3.7°, range 2-7°). Of those six, three stopped briefly during the 6minWT. On average, those six participants also tended to have a more severe degree of foot drop at the start of the 6minWT (4° of plantar flexion) compared to those who did not (2° of dorsiflexion). Twelve of the fifteen participants showed a decrease in cadence over the duration of the 6minWT (Table 3).

## 4. Discussion

The main purpose of the present study was to explore whether an adapted 6minWT would lead to a decrease in ankle dorsiflexion, suggesting motor fatigability, in people with MS with a clinical diagnosis of foot drop.

It was found that the average decrease in peak dorsiflexion in swing of 1.5 degrees was statistically significant and that six of the fifteen participants had a decrease of 2 degrees of more, which can be regarded as clinically significant [29].

Our finding that not all participants exhibited a deterioration in gait kinematics over the duration of the walking test agrees with the findings of Sehle et al. [20]. Using a preestablished cut-off value, based on their Fatigue index Kliniken Schmieder (FKS) derived from gait kinematics, these authors reported that just over 70% of their participants exhibited motor fatigue over an exercise task which involved walking until a RPE of 17 (‘very hard') was achieved or until 60 minutes had elapsed.

The participants in the study by McLoughlin et al. [21] underwent computerised 3D gait analysis of three walking trials just before and after a 6minWT. In the trials directly following the 6minWT, statistically significant decreases in peak dorsiflexion in swing and the angle ankle at initial contact were observed. Interestingly, although the average change in dorsiflexion reported was similar to that in our study (1.5 degrees), participants in Mcloughin's study [21] exhibited considerably less foot drop before the 6minWT (mean of 8° of peak dorsiflexion in swing, compared to 2° of plantar flexion in our study).

The majority of the studies assessing motor fatigability in people with MS have focused on the decrease in walking speed [15–18] over a set distance or time. In a large multicentre trial (n=208), Leone et al. [18] found that the majority of participants (EDSS 3.5-5.5) showed a continuous decrease (more than 5%) in walking speed over the duration of the 6minWT (31 out 54 participants (57%) and 19 out of 29 (66%) for EDSS 3-4 and EDSS 4.5-5.5, respectively). Phan-Ba et al. [16] recorded the time required to cover 100m during a 500m walk test in a large number of pwMS with EDSS ranging from 0 to 6. Compared to the first 100 m, pwMS with EDSS <4 slowed down only slightly more than the healthy controls (3-4 s versus 2 s); however the group with EDSS 4-6 was 15 seconds slower in their last 100m lap compared to the first lap. Finally, Dalgas et al. [17] showed that the first minute of the 6minWT was fastest and followed by a stable walking speed for people mildly affected by MS but a continuous decline during minute 2-5 for those moderately affected by MS.

Although we did not directly record walking speed during the 6minWT, our findings of a reduced cadence and increased duration of the stance phase during the last 10 gait cycles of the 6 minute walk also suggested a decrease in walking speed in 12 of the 15 participants.

In contrast, the participants in the studies by McLoughlin et al. [21] and Barr et al. [22] did not show a decrease in walking speed. The fact that these studies recorded the walking speed directly before and after, rather than during the 6minWT, and their inclusion of participants whose walking ability was only mildly impaired (average walking speed 1.16 m/s versus 0.77 m/s in our study) could explain the comparative discrepancy in results.

Compared to studies quantifying fatigability by a decrease in walking speed, we believe the strength of our study and those studies by McLoughlin et al. [21] and Sehle et al. [19, 20] is the quantification of fatigability at the ICF activity level using gait kinematics before and after or during a fatiguing task rather than walking speed. A focus on gait kinematics may potentially provide more insight into the mechanisms involved with motor fatigability as walking speed during prolonged walking can be influenced by many other factors such as pacing strategies [17] and aerobic fitness.

The main limitation of our study and the majority of other studies investigating motor fatigability in people with MS are the use of one set exercise task (i.e., 6 min walk test) for all participants irrespective of their walking capacity. It is possible that tests with longer duration and/or a higher intensity more closely resembling tasks in activities of daily living would have elicited a decrease ankle dorsiflexion in more participants. Further, the test- retest reliability of the change in ankle kinematics over the duration of a 6minWT was not assessed. This lack of information regarding the test-retest reliability of motor fatigability measures was highlighted in a recent systematic review [14] and should be addressed in future studies.

Another potential limitation of our study is the relatively small number of participants. However, the statistical power of our study was sufficient to detect statistically significant differences in temporal stride parameters and a relatively small mean change (1.5 degrees) in ankle dorsiflexion. A larger sample may have resulted in more statistically significant changes but these changes may have not been necessarily clinically significant.

Further, for safety reasons, we allowed our participants to stop and rest before continuing the 6minWT. It is possible that this allowed those participants to recover and thus show no or a reduced decrease in ankle dorsiflexion indicating motor fatigability. However, all three participants who stopped showed a decrease in dorsiflexion in swing ranging from 1.5 to 3.5° at the end of the 6minWT.

Other limitations include the use of a single ankle electrogoniometer; hence we were not able to ascertain whether motor fatigability, as defined as a deterioration in gait kinematics, is also apparent in joints other than the ankle and in the contralateral leg. Although the participants had a clinically diagnosed foot drop in only one leg, it is possible that participants also had a degree of neurological deficit in the other leg, negatively affecting the participant's gait characteristics such as stride time over the duration of the 6minWT. Similarly, impaired core stability and balance deficits could have become more prominent at the end of this task which involved four 90-degree turns over a distance of 32m. However, the focus of this study was on ankle kinematics in swing which may be less likely to be influenced by factors such as impaired balance and neurological deficits of the contralateral leg.

Finally, our study did not include an age matched control group so that we cannot be sure that the observed changes in gait pattern over the duration of the 6minWT are not also present in aged matched people without MS.

Future studies should address these aforementioned limitations in order to obtain a better understanding of the onset or worsening of foot drop after prolonged exercise as a sign of motor fatigability in people with MS.

## 5. Conclusion

The aim of the present study was to investigate whether an adapted 6minWT protocol would result in a decrease in ankle dorsiflexion as an outcome of motor fatigability in pwMS with clinically diagnosed foot drop. We found an average decrease in dorsiflexion of 1.5 degrees which was statistically significant and that 6 of the 15 participants showed a clinically significant decrease of 2 degrees or more.

Our results suggest that with the protocol used in this study it is feasible to identify patients who experience increased foot drop as a result of a prolonged exercise task without the need for a computerised 3D gait analysis system. Our observations have implications for clinical practice with respect to both the diagnosis of foot drop and the assessment of the efficacy of interventions treating foot drop in those patients whose ankle kinematics over a short distance are not or minimally affected. It is recommended that future studies assess the test-retest reliability of measures of motor fatigability.

## Figures and Tables

**Table 1:** Participant (n=15) characteristics, objective, and self-reported walking performance.

	**Mean ± SD (range)**
**Age in years ± SD (range)**	54.9 ± 7.7 (37 – 64)
**Males: females**	5: 10
**EDSS range**	4-6
**Type of MS (RR, PP, SP)**	4/7/4
**FES/AFO/none**	10/2/3
**MSWS-12 (range 0-100)**	72 ± 15 (50-90)
**6minWT (m)**	263 ± 79 (107-391)
**Walking speed (m/s) during 6minWT** **∗**	0.77(0.20)

*∗*n=12; excluding the three participants who stopped during the 6minWT.

**Table 2:** Means (SD) of the gait characteristics and RPE in the first and last 10 gait cycles of the 6minWT.

	**First 10 gait cycles **	**Last 10 gait cycles **	**p-value**
Ankle angle at IC (°)	-7.6 (4.6)	-9.0 (6.2)	0.13
Peak dorsiflexion in swing(°)	-0.4 (7.6)	-2.0 (8.7)	0.03
Cadence(steps/min)	49(6)	46(7)	<0.001
Timing peak plantar flexion (%GC)	66 (2)	69(3)	0.02
RPE (range 6-20)	7.9 (1.8)	12.1 (2.4)	<0.001

IC=initial contact; %GC=% gait cycle duration; RPE=rate of perceived exertion.

**Table 3:** Individual results for all participants.

Participant	MSWS12	6minWT (m)	DFswing(°) first 10 GC	Change DFswing(°)	Change in cadence (steps/min)
1	80	197	0.3	-3.7	-4
2	90	224	-4.9	1.6	-4
3	80	326	1.7	2.1	-5
4	70	107	-11.8	-1.8	-2
5	63	333	12.8	-0.8	-3
6	65	391	-0.6	-2.1	-1
7	73	191	10.8	-0.6	-8
8	87	216	10.8	1.3	-3
9	73	309	1.3	-1.5	0
10	90	179	-5.6	1.1	-5
11	82	295	-1.1	-6.7	-1
12	67	262	3.0	-1.9	2
13	50	263	-12.2	-3.3	-5
14	85	266	-4.1	-2.3	0
15	68	386	-6.5	-4.6	-2

## Data Availability

Any data presented in this article is available upon request from the corresponding author.
